# Studies of neurodegenerative diseases using *Drosophila* and the development of novel approaches for their analysis

**DOI:** 10.1080/19336934.2022.2087484

**Published:** 2022-06-29

**Authors:** Yohei Nitta, Atsushi Sugie

**Affiliations:** Brain Research Institute, Niigata University, Niigata, Japan

**Keywords:** Drosophila, neurodegenerative diseases, reverse translational research

## Abstract

The use of *Drosophila* in neurodegenerative disease research has contributed to the identification of modifier genes for the pathology. The basis for neurodegenerative disease occurrence in *Drosophila* is the conservation of genes across species and the ability to perform rapid genetic analysis using a compact brain. Genetic findings previously discovered in *Drosophila* can reveal molecular pathologies involved in human neurological diseases in later years. Disease models using *Drosophila* began to be generated during the development of genetic engineering. In recent years, results of reverse translational research using *Drosophila* have been reported. In this review, we discuss research on neurodegenerative diseases; moreover, we introduce various methods for quantifying neurodegeneration in *Drosophila*.

## 1. Introduction: Cross-species homology

The Nobel Prize in Physiology or Medicine 2021 was awarded to David Julius and Ardem Patapoutian. They identified transient receptor potential (TRP) and PIEZO-type mechanosensitive channel components (PIEZO) as proteins that are sensitive to temperature and mechanical stimuli. These temperature and mechanical sensors are conserved in *Drosophila* [1-3]. Before the human genome was sequenced, the *Drosophila* genome was read in 2000 [4]. Afterwards, the fly genes were compared with the human genes known by that year. Among the 289 genes involved in human diseases, Rubin et al. identified 177 orthologous genes (61%) [5]. Reiter et al. also reported 714 *Drosophila* orthologs in 929 human disease genes (77%) described in the Online Mendelian Inheritance in Man (OMIM) [6]. Furthermore, human orthologous genes in *Drosophila* are reportedly highly conserved among the genes essential for survival [7]. In this report, ethyl methanesulfonate (EMS), a chemical mutagen, was used to induce random mutations in *Drosophila* chromosomes. The lethal genes were screened on a large scale. Of 165 genes identified for survival, 93% (153 genes) were orthologous to human genes. This suggests that genes required for survival are evolutionarily conserved between humans and flies.

This review focuses on studies and evaluation systems of neurodegenerative diseases in *Drosophila*, a simple model organism.

## 2. Drosophila used as a model for studying neurodegenerative diseases

*Drosophila* has several advantages as a simple model organism. First, it can undergo rapid genetic analysis because of the relatively few duplicated genes in its genome, thereby allowing for genetic analysis without considerable functional redundancy. It is also important because of its small size, low cost, ease of rearing, and short life cycle. Second, *Drosophila* has a compact brain, which is advantageous in research on neurodegenerative diseases. The *Drosophila* brain consists of neurones and glial cells, whose functions are similar to those of vertebrates. For example, the visual system circuit in vertebrates and flies uses common design principles and has many similarities in neurobiology, including neurotransmission, synaptic plasticity, and neurogenesis [8,9]. Interestingly, in contrast to vertebrates, the photoreceptor in *Drosophila* is depolarised by light. Since this change in membrane potential can be easily monitored, the *Drosophila* photoreceptor is a powerful genetic model for neuronal structure and function. Age-related decline in neural performance is also seen in *Drosophila* as motor function decreases with ageing in flies [10,11]. Age-dependent memory decline is also seen in *Drosophila* [12,13]. The olfactory function of flies declines with age. Within the olfactory circuit, cholinergic projection neurones showed reduced olfactory responses with loss of axonal integrity and decreased synaptic marker proteins [14]. Thus, *Drosophila* is valuable for research on ageing. Moreover, a single-cell transcriptomic atlas of the entire adult *Drosophila* brain sampled over a lifetime has been provided [15]. This outcome can comprehensively search all transcriptional states throughout the ageing brain. Ageing is an important risk factor for many neurodegenerative diseases, including Alzheimer’s disease (AD) and Parkinson’s disorder (PD). Thus, similarities can be found in the structure, function, and ageing between the fly and the human brain.

*Drosophila* has long been a simple model organism that can undergo easy genetic analysis. The discovery of various mutants and the identification of the responsible genes for neurodegenerative diseases have often been achieved for the first time in *Drosophila*. Hitherto, there has been an increase in the knowledge on the gene regulation of the nervous system. Therefore, genetic and functional analyses that have been discovered in *Drosophila* in the past can provide insights into research that will reveal pathogenic mechanisms of disease in later years. For example, the genes *Shaker* (*Sh*) and *ether a go-go* (*eag*) were reported more than 50 years ago as mutants in which legs shake abnormally under moderate ether anaesthesia [16]. The phenotypes of these mutants suggest that their related genes play important roles in neuronal function. Gene cloning has been performed and two families of potassium channels have been identified [17–19]. *Sh* is the first potassium channel gene to be identified in all organisms, thereby making it possible to identify biochemical purification and molecular characteristics of vertebrate potassium channels [17]. Another potassium channel family was identified by cloning and sequencing *eag*. Based on the homology of this sequence, a mutation of *human ether-à-go-go-related gene* (*HERG*) in patients with Long QT syndrome has been reported [20]. Several families of potassium channels are involved in many neurological disorders, including benign familial neonatal convulsions, hereditary deafness, neurodevelopmental disorders, and neurodegeneration [21,22]. Thus, studies that uncover novel genes and fundamental biological phenomena using flies may reveal the key pathways for molecular pathogenesis.

Research on neurodegenerative diseases using flies has developed gradually with the progress of technologies in both clinical and basic research fields. From the 1990s to the beginning of the 2000s, when genetic engineering technology emerged, disease models of *Drosophila* were beginning to be created based on knowledge obtained from human diseases. The expression of human pathogenic proteins in flies also exhibited toxicity. This indicated a conservation of the molecular pathological mechanism and the potential for the use of flies as disease models. For example, neurodegenerative diseases are caused by the expansion of CAG repeats within the protein-coding region of a causative gene. Spinocerebellar ataxia type 3 (SCA3/MJD) is one of at least eight human neurodegenerative diseases caused by glutamine repeat elongation [23]. Segments of the SCA3/MJD protein have been used to reproduce glutamine repeat disease in *Drosophila*. The expression of human proteins containing extended polyglutamine repeats in flies resulted in the formation of nuclear inclusions and neurodegeneration similar to those in humans [24]. In addition, Jackson et al [25]. attempted to express the expanded CAG repeats in the huntingtin protein responsible for Huntington’s disease (HD), an autosomal dominant neurodegenerative disease, in the photoreceptor neurones of *Drosophila* by using *Glass Multiple Reporter (GMR)-Gal4* [26]. They found that polyglutamine-extended huntingtin formed intranuclear inclusions and induced neurodegeneration in the fly retina as in human neurones. Two years later, a large-scale modifier screen was searched for genes that could suppress Htt’s polyQ toxicity, taking full advantage of the fly’s rapid genetics. Expression of 127 polyQ in fly eyes with *GMR-Gal4* induces severely abnormal eyes. This abnormal eyes show that the disordered alignment and dysmorphic structure of the compound eyes that called the rough eye phenotype (REP) . This phenotype, easily identified under a standard microscope, was used as a pathological indicator. Therefore, in a later section, we describe the REP in more detail, ‘*2.1.1 Rough eye phenotype*’. The polyQ-induced REP fly was then mated with as many as 7000 P-element insertion strains. These P-element insertion strains were *de novo* generated EP collections in which the UAS element was inserted into the promoter region of the endogenic genes. Therefore, the strains were expected to overexpress genes. The extensive screening narrowed down the number of lines capable of suppressing the phenotype to 30 and enhancing the phenotype to 29. *Human DnaJ protein 1 (HDJ1*) and *tetratricopeptide repeat protein 2* (*dTPR2*) orthologues were identified as the strongest suppressors [27].

*Drosophila* models have also been established for PD, a neurodegenerative disorder characterised by dopaminergic neurone loss, Lewy body formation, and motor deficits in the substantia nigra [28,29]. Feany et al. reported that α-synuclein is expressed in different cell types [28]. For example, the Gal4 driver *embryonic lethal abnormal vision Gal4* (*Elav-Gal4*) [30] has been used to express α-synuclein wild type (WT), A30P, or A53T mutations in all neurones. After 30 days, the cell bodies of dopamine neurones stained with tyrosine hydroxylase antibody disappeared, and the inclusion of α-synuclein was observed in the suboesophageal ganglia. Moreover, climbing ability, an index of motor function, dropped significantly from 3 weeks. Before the cell death, neural processes of dopamine neurones, in which α-synuclein was expressed in dopaminergic neurones using a driver line with the 3,4-dihydroxyphenylalanine (DOPA) decarboxylase gene promoter (*Ddc-GAL4*) [31], disappeared after 10 days. In the other tissue, the expression of α-synuclein in the eye with *GMR-Gal4* resulted in the observation of retinal degeneration after 30 days [28]. Auluck et al. also confirmed LB-like inclusion when α-synuclein was expressed in dopaminergic neurones using *Ddc-Gal4*, and the number of anti-TH-positive dopamine neurones decreased by 10 days [29].*Vacuolar protein sorter-35* (*VPS35*) encodes a subunit of the retromer complex whose mutations cause late-onset PD [32,33]. The retromer is essential for sorting and recycling specific cargo proteins from endosomes to the trans-Golgi network and cell surface. Retromer complexes are highly conserved, and orthologous genes have been found in yeast, worms, flies, mice, and humans [34]. Whole-nerve knockdown of *Drosophila* VPS35 (*dVps35*) by *Elav-Gal4* driver impaired retrograde transport from the endosome to the trans-Golgi by retromers, thereby impairing lysosomal degradation of human α-synuclein expressed in the *Drosophila*. As a result, it increased the number of α-synuclein inclusions and impaired motor function [35]. Another study showed that *Drosophila* eyes induced loss-of-function *dVps35* mutant clones resulted in ceramide accumulation. Loss of retromer function causes accumulation of ceramides and sphingolipid intermediates, leading to retinal degeneration [36]. In many types of PD, changes in endolysosomal function and sphingolipid metabolic pathways may be affected. Other possible pathologies have also been suggested. *dVps35* controls the recycling of synaptic vesicles through the endosomal pathway. In the genetic background of a *dVps35* mutant, *dVps35* D647N, a mutation involved in PD, cannot be rescued. This implies that loss of *VPS35* function, preventing synaptic vesicle recycling, is an important aspect of the pathogenesis of PD [37].

*Parkin* functions as an E3 ubiquitin protein ligase [38–40]. A deletion mutation in *Parkin* has caused autosomal recessive juvenile parkinsonism in humans [41]. A *Parkin* ortholog also exists in *Drosophila, dParkin*. Greene et al. generated the null mutants for analysing *dParkin* function. The mutants had a shorter lifespan and reduced locomotor function. These were derived from the apoptosis of muscle tissue. In muscle tissue, swollen mitochondria showed severe disruption and cristae disintegration. On the contrary, no obvious degeneration of dopaminergic neurones was observed. This differs from the anatomical condition of PD in which dopaminergic neurones in the substantia nigra degenerate. However, mitochondrial dysfunction, the underlying molecular mechanism responsible for pathology in these different tissues, may be highly conserved [42]. The damaging effect of human α-synuclein can be suppressed by expressing the fly *Parkin* [43,44]. This suggests that up-regulation of *Parkin* may suppress the α‐synuclein pathology of PD.

*GBA1* encodes the lysosomal enzyme acid-β-glucocerebrosidase (GCase). Gaucher disease (GD) is a lysosomal storage disease caused by mutations in the *GBA1* gene [45]. Parkinsonism was found in some patients with GD in 1996 [46]. *Drosophila* has two *GBA1* ortholog genes, *dGBA1a* and *dGBA1b*. Mutants of these genes were created in 2016 to characterise the function of the *GBA1* ortholog [47]. Large, distorted lysosomes were observed in the brain of the *dGBA1b* mutant. Atg8a protein, an ortholog of microtubule-associated proteins 1A/1B light chain 3B (LC3) localised to the autophagosome membrane, was accumulated in the fly brain. These results indicate that *dGBA1b* mutants block lysosomal and autophagy functions. In addition, electron microscopy revealed that mitochondrial function was impaired due to increased mitochondrial size and decreased ATP levels. Mutants of *dGBA1b* had a shorter lifespan and decreased locomotor function in an age-dependent manner [47]. Knockdown of *dGBA1b* was found to promote aggregation of the triton-insoluble α-synuclein, indicating that the fly *GBA1* orthologous gene is useful for analysing human molecular pathology [48].

Progressive neurodegeneration was also observed by the expression of β-amyloid (Aβ) or *Tau* encoded by the gene microtubule-associated protein tau (MAPT), both of which are associated with the main pathologies of AD, in *Drosophila* brain [49–52]. AD is a progressive neurological disorder that causes irreversible loss of neurones, especially in the cortex and hippocampus. In the brains of patients with AD, senile plaques that contain Aβ and neurofibrillary tangles (NFTs), aggregates of highly phosphorylated Tau protein, are observed. These are the major pathologic features of AD [53]. *Drosophila* is widely used as a simple *in vivo* model for the molecular pathogenesis of AD [54,55]. Among the various *Drosophila* models, flies that express Aβ are widely used disease models. Although there are no peptides homologous to Aβ in *Drosophila*, flies expressing human Aβ in all nervous systems have been shown to have impaired learning, accumulation of amyloid plaques, neurodegeneration, and shortened lifespan, as observed in patients with AD [49–51].

Whole-neuronal expression of *Tau* using *Elav-Gal4* was short-lived and increased the number of vacuoles in the cortex and neuropile of flies with age [52]. The R406W mutation, the cause of frontotemporal dementia with Parkinsonism linked to chromosome 17 tau with AD-like clinical features [56–58], was more severe in Tau-related pathology [52]. In addition, the expression of *Tau* in cholinergic neurones by *Choline acetyltransferase-Gal4* (*Cha-Gal4*) [59] dramatically reduced the number of cholinergic neurones after 60 days [52]. Cholinergic neurones are particularly vulnerable to neurodegeneration in AD and can be affected in tauopathies [60].

Strong risk factors for AD include advanced age and having at least one *Apolipoprotein E* (*APOE*) *ε4* allele [61]. The *APOE* gene encodes the ApoE protein and is mainly produced by astrocytes and activated microglia in the brain [62]. Carrying the *APOEε4* allele increases the risk of dementia by 3 ~ 4 fold in heterozygotes and 12 ~ 15 fold in homozygotes compared with *APOEε3* carriers [61]. *Drosophila* does not have direct *APOE* orthologs, but has *APOD* orthologs, *Glial Lazarillo* (*Glaz*) and *Neural lazarillo* (*Nlaz*). The knock-down of *Glaz* in glia by *54C-Gal4* prevents the lipid droplet formation formed by the elevation of reactive oxygen species (ROS), causing retinal degeneration in the fly eye. This was reversed by the expression of human *APOEε2* and *APOEε3* in fly glia, but not *APOEε4* [63]. This suggests that *Glaz* and human *APOE* can replace function. Importantly, *APOEε4* failed to recover. From this fact, the failure of lipid droplet formation from lipid transport between neurone and glia may be a part of the pathological mechanism of AD.

Amyotrophic lateral sclerosis (ALS) is characterised by progressive degeneration of motor nerve cells in the brain (upper motor neurones) and spinal cord (lower motor neurones). *Cu/Zn-superoxide dismutase* (*SOD1), TAR DNA-binding protein of 43kDa* (*TDP-43), fused in sarcoma/ translocated in liposarcoma* (*FUS/TLS)*, and *vesicle-associated membrane protein-associated protein B* (*VAPB*) are genetic factors in patients with ALS. Since around 2010, flies have been used to analyse molecular pathological mechanisms associated with these risk genes.

*Drosophila* can easily induce any gene expression, specifically in motor neurones. The *D42-Gal4* driver [64] was used to express the human *SOD1* WT, A4V [65] and G85R mutations [66] found in patients with ALS, in the motor neurones of flies, as an attempt to reproduce a part of ALS disease symptoms in flies [67]. Human *SOD1* WT, A4V, and G85R mutants had significantly worse motor function with the climbing assay after 28 days. In electrophysiological experiments, the response of dorsal longitudinal muscles decreased in G85R mutant and WT, and the response of tergotrochanteral muscles decreased slightly in G85R mutant. This leads to a progressive loss of synaptic transmission in flies expressing human *SOD1*. Human *SOD1* G85R accumulated in the cytoplasm of motor neurones as foci. This study showed the effect of *SOD1* on fly motor neurones. Although *SOD1* expression in motor neurones alone was not short-lived and did not result in a decrease in cell number due to neurodegeneration, the pathological findings indicated a reduction of synaptic transmission, a foci-like accumulation of *SOD1*, and a decrease in locomotor activity, which were considered presymptomatic [67].

In the case of TDP-43, a strong phenotype was observed with a short life span [68], and the number of synaptic boutons and branches decreased in the neuromuscular junction (NMJ), as did the locomotive activity [69]. In addition, the molecular pathophysiology in which cytoplasmic TDP-43 is toxic was clarified [70]. In that research, the expression of human *TDP-43* in the eye, which cannot localise to the nucleus, results in REP. Antibody staining also confirmed that the *TDP-43* M337V mutation, in which the human sporadic ALS mutation occurred [71], also showed REP and mislocalisation of TDP-43 occurring in the nucleus and the cytoplasm.

The RNA/DNA-binding proteins FUS (also known as TLS) is also known to link to familial ALS and frontotemporal dementia (FTD), such as TDP-43 [72,73]. Expression of WT *FUS* in *GMR-Gal4* did not result in rough eyes, but R518K, R521H, and R521C mutations caused REP. Using a GeneSwitch system that can induce tissue-specific gene expression by feeding the drug RU486 (also known as Mifepristone) [74,75], the expression of *FUS* in all nervous systems from the adult stage was shown to increase locomotor defects and mortality [76]. In that study, expression of *FUS* by *ok371-Gal4* [77] in motor neurones also resulted in larval-crawling defects in locomotor activity. FUS is a nuclear and cytoplasmic shuttle protein predominantly located in the nucleus. The deletion of the nuclear export signal (ΔNES) rescued toxicity associated with mutant *FUS*, suggesting that cytoplasmic localisation of mutant *FUS* is required for causing ALS pathogenesis. Furthermore, expression of both *FUS* and *TDP-43* in the eyes with *GMR-Gal4* promoted more severe rough-eye by synergic stimulation [76]. The REP was also induced when ALS associated R524S or P525L mutation [72,73] was expressed in the eye using *GMR-Gal4*, and the localisation of mutant *FUS* was remarkable in the cytoplasm by expression in motor neurones using *ok371-Gal4* [78]. Thus, it was clarified that *FUS* mutation has pathological significance for ALS in flies. Moreover, mutants of *cabeza* (*caz*), the fly *FUS* ortholog, are less than 20% eclosion capable but are rescued by expressing human *FUS*, indicating that function is conserved [79]. It was also able to rescue locomotion activity, but the rate of rescue was lower for the pathologic mutations R522G and P525L. This evidence suggests that the R522G and P525L mutations result in the loss of at least some activities of the FUS protein [79].

Mutations in human *VAMP-associated protein B* (*hVAPB*) are responsible for the occurrence of ALS 8, a type of ALS. In the *hVAPB* amino acid sequence, the region containing a P56S mutation was also conserved in the fly homologue *DVAP*, corresponding to a P58S mutation. The expression of the P58S mutation of *DVAP* in the *Drosophila* central nervous system using *C164-gal4* causes *DVAP* to aggregate in the cytoplasm and induce ubiquitinated inclusion bodies [80]. These protein inclusions accumulated in the endoplasmic reticulum (ER) and caused structural changes. Furthermore, mutant *DVAP* induced Unfolded Protein Response (UPR) that was positive for the UPR marker Hsc3 in the brain upon expression by *Elav-Gal4*. This evidence also demonstrated important similarities with familial and sporadic cases of ALS in the *Drosophila* model, including cytoplasmic inclusions, ubiquitination, and UPR [80]. Ubiquitin-positive aggregation was also observed in larval NMJ when *DVAP* P58S was expressed in muscle using *G14-Gal4* [81]. In the genetic context of loss-of-function in the *DVAP* mutant, expression of human *VAPB* in neurones can rescue the lethality associated with *DVAP* loss-of-function mutations, NMJ morphology, and electrophysiologically increase the mean frequency of miniature excitatory junctional potentials (mEJPs) phenotype. These data indicate that *DVAP* and human *VAPB* perform homologous functions [82].

Thus, at the beginning of the 21st century, *Drosophila* models expressing human disease-causing genes were shown to mimic the essential features of human disease, and therefore provided a powerful genetic approach to various neurodegenerative diseases such as polyglutamine diseases including HD, PD, AD, and ALS.

## 3. Reverse translational research on neurodegenerative diseases using Drosophila

**Figure 1.**
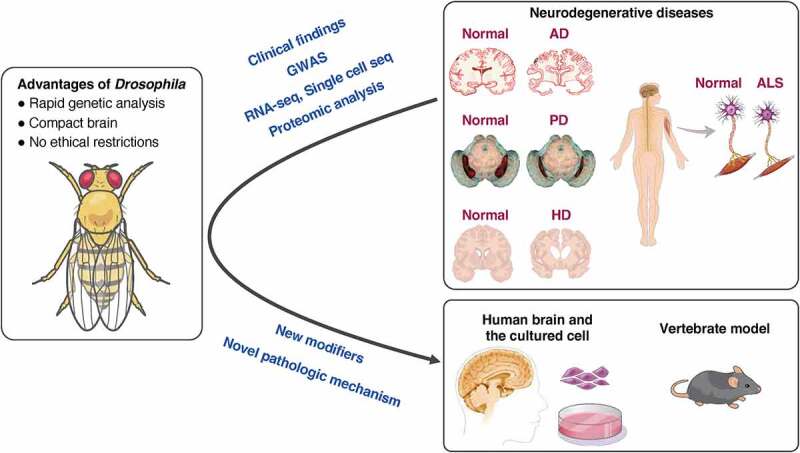
Reverse translational research using *Drosophila.*

Over the past two decades, a number of pathological mechanisms of various neurodegenerative diseases have been proposed based on studies using flies. The boundary between clinical and basic research has gradually diminished. In recent years, results of reverse translational research have been reported wherein novel molecular pathologies were discovered in flies based on knowledge obtained from human diseases; these findings were then directly demonstrated in human tissues (Figure 1). We begin this section with the findings from human genetic analyses that allowed us to estimate the genetic and molecular pathways involved in neurodegenerative diseases. Then, we will focus on reverse translational research, in which the discovery of new pathologies and relevant factors in flies was made based on the findings obtained in human clinical and research studies, and the findings were confirmed in human brain tissue (Table 1).

**Table 1.** Example of reverse translational researches.

Disease	Fly model	Novel processes or modifiers identified in fly	Patient tissue	Reference No.
LBD	Panneuronal OE of αsynuclein	abnormalities in the actin filament network and the formation of rod-shaped actin-positive structures in neurones	The formation of rod-shaped actin-positive structures in cingulate cortex	101
AD and PSP	Panneuronal OE of Tau	relaxed heterochromatin	The increased transcription of genes and transposable elements in post-mortem brains	102, 103, 104
AD	Panneuronal OE of Tau	Deficient in endogenous biotin	The reduced carboxylase biotinylation in frontal cortex	105
ALS	eye OE of TDP-43	ATXN2	The accumulation of ATXN2 in the cytoplasm of motor neuronse	109
ALS	eye OE of TDP-43	ALYREF	Elevated ALYREF in the motor neurones	114
ALS	eye or panneuronal OE of DVAP	Rab5	The accumulated RAB5 in the cytoplasm in motor neurones	115
FTD-ALS	eye OE of G4C2 repeats	PAF1 complex	The elevated PAF1 mRNA in the frontal lobes	111
FTD-ALS	eye OE of G4C2 repeats	dIF4B and eIF4H	The down-regulated eIF4H in the post-mortem brains	112

Genetic risk factors for AD have been reported to include rare dominant mutations in amyloid protein precursor (*APP*), presenilin-1 (*PSEN1*), presenilin-2 (*PSEN2*) and more common but incomplete penetrance mutations, such as *APOE* [83]. In addition, large patient cohorts have become available due to technological advances in next-generation sequencers, enabling unbiased genome analysis. This identified rare variants in the *sortilin related receptor 1* (*SORL1), ATP binding cassette subfamily A member 7* (*ABCA7*), and *triggering receptor expressed on myeloid cells 2* (*TREM2*) genes [84–87]. More recently, a large genome-wide association study (GWAS) of over 400,000 clinically-diagnosed AD, AD-by-proxy, and control cases were conducted to identify novel genetic mutations in AD. This meta-analysis identified 29 risk loci and identified 215 potentially causative genes. Of these, nine new loci have been identified [88]. The associated genes were strongly expressed in immune-related tissues such as the spleen, liver, and microglia. A genome-wide association meta-analysis was reported using more than 90,000 clinically diagnosed late-onset AD patients (LOADs) to identify LOAD risk loci in the same year. This analysis identified 25 loci, of which five new loci were identified [89]. Such large-scale GWAS analyses play an important role in suggesting significant disease mechanisms in AD and other diseases.

A recent GWAS analysis for PD identified 90 independent loci from 37,688 PD cases, 18,618 proxy-cases, and 1,417,791 controls. Of these, 38 loci were new [90]. Whole-genome sequencing was performed in 2,591 patients with Lewy body dementia (LBD) and 4,027 controls. The analysis identified five independent risk loci. These loci contained *GBA1, APOE*, and *SNCA*, respectively, which are known LBD risk loci. The two new loci contain *bridging integrator 1* (*BIN1*) and *transmembrane protein 175* (*TMEM175*). Interestingly, *BIN1* is a possible risk gene for AD, and *TMEM175* is associated with PD, suggesting common pathophysiology between neurodegenerative diseases [91]. In ALS, a GWAS involving 29,612 patients and 122,656 controls identified 15 risk loci [92]. The scale and number of GWAS analyses are increasing yearly. Further updates are expected to improve the accuracy of pathologic mutation detection.

The findings of meta-analyses obtained from humans, including the GWAS analysis described above, are powerful in analysing the molecular pathology of neurodegenerative diseases. Large-Scale GWAS, transcriptome-wide association studies (TWAS), proteomic, and metabolomic analyses of AD have comprehensively summarised the evidence of major cellular/molecular pathways in AD as a whole in terms of (I) progression of the Aβ pathway, (II) inflammatory/immune responses, (III) lipid homeostasis, (IV) regulation of endocytosis and vesicle-mediated transport, (V) regulation of the cell cycle, (VI) oxidative stress response, and (VII) axonal guidance [93]. For PD, ALS, and HD, a number of multi-omics analyses, including transcriptome, proteome, and metabolome analyses, have been conducted to identify pathogenic cell types and biological pathways, in addition to the identification of risk loci by GWAS. Therefore, the prediction of genes and molecular pathways involved in the disease has become possible [94–96].

To determine whether a candidate gene or molecular pathway contributes to the disease state, it is necessary to functionally validate using model organisms. Given the multitude of candidates to investigate, a simple model organism, *Drosophila*, can quickly and easily assess the pathological significance of a target gene mutation and analyse the disruption of the molecular pathways involved in the disease. For example, candidate genes for AD-related genomic loci based on GWAS were tested to determine whether they were modulators of Tau-induced neurodegenerative disease, and 15 genes were identified [97,98]. In those studies, expression of the *Tau* V337M mutation under the control of *GMR-Gal4* led to a simple screen with a phenotype that caused moderately reduced eye size and a roughened surface [99]. This experimental system was also used in another study in which four homologues, *Cas scaffold protein family member 4* (*CASS4), EPH receptor A1* (*EPHA1), protein tyrosine kinase 2 beta* (*PTK2B*), and *MAP kinase activating death domain* (*MADD*), were newly identified as modulators of Tau toxicity [100], making it ideal to conveniently test genetic interactions with *Tau*.

Thus, there are increasing amounts of genetic and molecular information available from humans. The benefits of studying diseases using flies are being recognized immensely. Flies allow for the dissection of the aberrant biology underlying associated neurodegenerative disease.

Synucleinopathies, such as PD and LBD, cause damage to neurones via the aggregation and deposition of α-synuclein protein. One of the mechanisms responsible for the toxicity of α-synuclein was identified in the Feany laboratory. In Ordonez et al [101]., the authors found that the expression of α-synuclein in *Drosophila* brain resulted in abnormalities in the actin filament network and the formation of rod-shaped actin-positive structures in neurones. This was also observed in the post-mortem brain of a patient having dementia with Lewy bodies and in a synucleinopathy mouse model. This is an innovative reverse translational study in which a new pathological mechanism was discovered in flies, and it was confirmed that the pathological features were conserved in the human brain and mouse brain.

Patients with tauopathies, including AD, familial frontotemporal lobar degeneration (FTLD), and progressive supranuclear palsy (PSP), exhibit pathological features in which the microtubule-associated protein Tau is fibrillated and deposited in neurones. One of the various pathological mechanisms induced by Tau is the oxidative stress-induced DNA damage, which leads to neurodegeneration. Studies investigating the effects of Tau on the nucleus have found that heterochromatin is relaxed in the tauopathy model of *Drosophila* expressing the R406W mutation in *Tau*, which causes FTLD in the AD model of mice and in the brains of patients with AD. These findings indicate an increased transcription of genes and transposable elements that should be inactivated in heterochromatin, not only in the fly model but also in patients with AD and PSP [102,103]. RNAseq data from the brains of 636 patients with AD also revealed that the transcription of retrotransposon elements was activated [104]. Moreover, this study demonstrated that the expression of human *Tau* in *Drosophila* activates endogenous transposons in flies, indicating the conservation of pathological mechanisms due to chromatin relaxation.

In another study, modifier factors were identified from extensive genetic screening [105]. Modifiers are factors that promote or suppress pathological conditions caused by disease-associated proteins. In this study, human *Tau* was expressed in all neurones using the Gal4 driver *Elav-Gal4*. In addition, the signal of the caspase reporter *CD8-PARP-Venus* [102,106] was used as an indicator to test as many as 7,204 RNAi lines. The identified modifiers were 63 suppressors and 306 enhancers. By applying these orthologous human genes to the STRING interactome generator [107], enriched pathways such as metabolic and mitochondrial pathways, known tau-mediated neurodegenerative mechanisms, were obtained. Among metabolic pathways, the knockdown of the *Biotinidase* (*Btnd*) gene increased neurotoxicity. Biotinidase releases B-vitamin biotin from histone and carboxylase degradation products. As B-vitamin can be administered orally, its potential therapeutic effects as a supplement were considered, further exploring the effects of the biotin metabolic pathway. Flies expressing *Tau* were found to be deficient in endogenous biotin due to chromatin relaxation. Carboxylase biotinylation was also found to be reduced in the frontal cortex of patients with AD. Fly-specific studies, which allow high-throughput gene exploration, have shown that biotin deficiency can affect neuronal health.

ALS is a severe neurodegenerative disease involving the loss of motor neurones in the cerebral cortex and spinal cord. TDP-43 is known to play a primary role in the pathogenesis of ALS [108]. Yeast and flies were used to explore the factors that suppress or promote TDP-43-induced toxicity [109]. First, a large-scale screening of 5000 genes was performed using yeast to alter the toxicity of TDP-43 using yeast via the expression of both endogenous yeast protein and human *TDP-43*. Thus, Pab1-binding protein 1, an orthologue of human ATAXIN 2 (ATXN2), was identified as a candidate protein for promotion of the TDP-43-induced toxicity. Thereafter, ATXN2 was confirmed using the REP of *Drosophila* to determine whether it promotes TDP-43 pathology. The toxicity of etiologic factors such as TDP-43 causes structural abnormalities in the compound eye by expression of human *TDP-43* with *GMR-Gal4 driver. ATXN2* orthologue knockdown in *Drosophila* eye suppressed the toxicity of TDP-43, and overexpression of the *ATXN2* orthologue in the eye resulted in more severe compound eye defects. Thus, the candidate pathological modifier, *ATXN2*, was identified and validated from yeast cells and flies. Finally, it was found that ATXN2 strongly accumulated in the cytoplasm of motor neurones of patients with ALS.

A common genetic factor in patients with ALS and frontotemporal dementia (FTD) is the extension of the GGGGCC repeat within intron 1 of Chromosome 9 open reading frame 72 (C9orf72) gene. Dipeptide repeats (DPR) expressed in this sequence are also toxic in *Drosophila*, causing compound eye defects and decreased survival [110]. To address this DPR toxicity, the Bonini group identified a novel PAF1 complex [111] as well as *eIF4B* and *eIF4H* as disease modifiers [112]. The screening in which *PAF1* was identified was as follows. Using the rough eye induced by *GMR-Gal4* expression of (G4C2)_49_ repeats as an indicator, an unbiased RNAi screen was performed, which was eventually crossed to 3,932 RNAi lines, resulting in 55 suppressor and 64 enhancer genes. For these 119 modifiers, gene ontology term analysis was performed to determine enriched processes, with strong enrichment for genes associated with RNAP II-driven transcription, including components of the PAF1 complex. Thus, the PAF1 complex is a transcriptional regulator that suppresses the toxicity of (G4C2)_49_ repeats. For *eIF4B* and *eIF4H*, the following screening was performed to identify key factors underlying G4C2-related repeat-associated non-ATG translation (RAN) translation. To address this, flies expressing expanded G4C2 repeats by *GMR-Gal4* were crossed with 48 lines of either the RNAi or loss-of-function lines to obtain suppressors and enhancers. It included 48 of 56 translation factors known to exist in the *Drosophila* genome [113]. Finally, 11 RAN-translation factors were selected, including *eIF4B* and *eIF4H*. Further analysis showed that the transcription level of *PAF1* mRNA was elevated in the frontal lobes of patients with FTD-ALS [111]. The expression of *eIF4H* was down-regulated in the post-mortem brains of patients with FTD-ALS having long GGGCC repeats [112]. The results obtained in flies were also reflected in humans.

RNA-binding proteins such as TDP-43 are involved in ALS and FTD pathogenesis. Thus, it is possible that there are other RNA-binding proteins involved in the pathogenesis. To address this issue, RNAi screening was performed in which 107 *Drosophila* genes with RNA recognition motifs were knocked down in the fly eye that expressed *TDP-43* or *TDP-43* and *ATXN2-32Q* by *GMR-Gal4*. The screen identified a total of 22 modifiers and *Ref1* was identified as the strongest hit [114]. The results showed that the toxicity of *TDP-43* expressed in compound eyes was suppressed by the knockdown of the mRNA export factor *Ref1*. The protein expression of the human orthologue *ALYREF* was elevated in the motor neurones of patients with ALS. Thus, *ALYREF* has been shown to increase the risk of FTD-ALS.

An analysis of the *Drosophila* orthologue *VAMP-associated protein 33kDa* (*DVAP*) revealed that one of the mechanisms of ALS 8 is associated with abnormal vesicle transport and endocytosis [115]. To identify the pathomechanism mediated by the P58S of *DVAP*, a genetic modifier screen was performed by overexpressing endogenous genes. When *DVAP* P58S was expressed in the eye using an *eyeless-Gal4* driver, the size of the eye was reduced to about 30% with a REP. A collection of EP and EPgy2 genome-wide insertion mutations was targeted for this eye phenotype. These collections consist of UAS elements inserted into the promoters of endogenous genes. Thus, GAL4-expressing cells can overexpress genes downstream of the UAS sequence [116]. A total of 1183 individual EPs or EPgy2 lines were crossed to determine whether the F1 progeny eye phenotype was suppressed or enhanced. As a result, 85 modifiers were identified, including 71 suppressors and 14 enhancers. Next, to examine whether candidate factors were involved in the motor system more closely related to the pathology of ALS, *DVAP* P58S was expressed by E*lav-Gal4*, a climbing assay verified the motor function, and the defectivity of motor neurones synapses was tested. The results showed that 42 out of 85 are modifiers that affect the function and structure of motor neurones. From these candidate genes, mechanisms regulating endocytic transport, proliferation, and apoptosis were deduced as potent modifiers of ALS8-mediated defects. Among them, *Rab5* was a strong modifier for the *DVAP* P58S phenotype, indicating that up-regulation of *Rab5* functions as a strong suppressor. Next, immunohistochemistry was performed in human postmortem spinal cord tissue to determine whether *RAB5* is present in human motor neurones and whether its localisation is affected in ALS. In the spinal cord tissue of patients with ALS, RAB5 GTPase, an early endosomal marker, was actually found to abnormally accumulate in the cytoplasm.

## 4. Quantitative methods in neurodegenerative disease studies in drosophila

**Figure 2.**
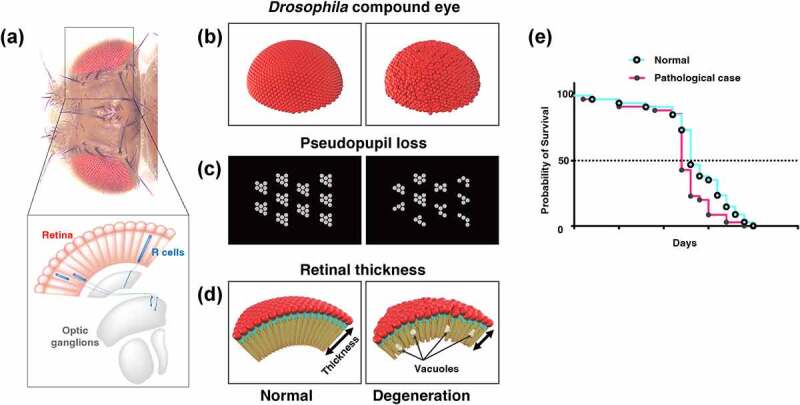
Traditional methods for evaluating neurodegeneration using *Drosophila*. (A) The dorsal view of the head of *Drosophila* and a cross-sectional view of the visual system. 700–800 ommatidia are aligned in the retina. Each ommatidium has 8 types of photoreceptors: R1-6 project their axons to the first optic ganglion lamina, and R7-8 project their axons to the second optic ganglion medulla to transmit light information to the brain. (B-D) Structural defects observed in *Drosophila* compound eye (B), pseudopupil loss (C), and the retinal thickness and vacuolization (D) provide simple methods for evaluating the cytotoxicity of disease-associated proteins. (E) Life span analysis. The Kaplan-Meier survival curve is used to compare the lifespan of different groups.

**Figure 3.**
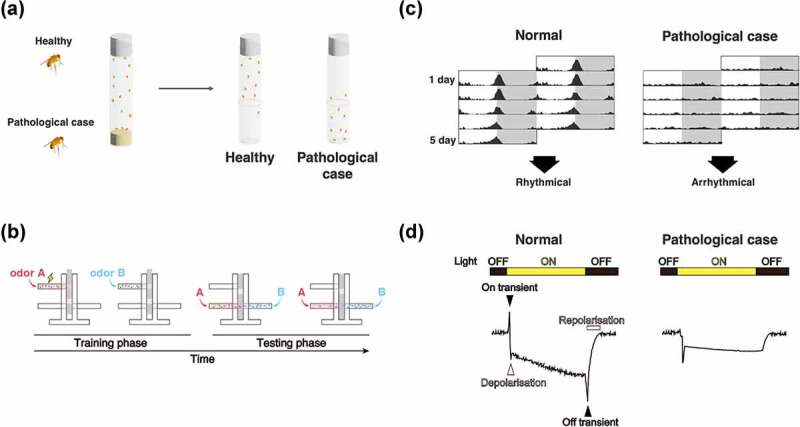
Evaluation methods of neural function using *Drosophila*. (A) Climbing assay. It is a measurement of motor function taking advantage of the fly’s negative geotaxis. (B) Olfactory memory assay using T-maze. At the training phase, unconditioned stimulus (US) such as electric shock and sweetness such as sucrose is associated with the odour in the training tube. Next, another odour is presented, without US. In the test phase, the two odours used in the training are presented to the trained fly from both ends of the test tube, and the performance index is quantified by which odour attracted the fly. In the figure, the associative learning between odour A and electric shock, called aversive learning, leads to the learning flies avoiding odour A. (C) Schematic diagrams of Actogram. Actogram is a double-plotted graphical representation of the phases of an organism’s daily activity and resting time. Grey shading indicates the dark phase. In controls, the level of activity increases in the morning and evening, but when the circadian rhythm is disrupted by the expression of disease gene, this time-specific increase is not observed. (D) Schematic diagrams of ERG trace. In healthy flies (left panel), the ERG traces are derived from photoreceptor activity (depolarisation and repolarisation, white arrow heads in the figure) and from postsynaptic neuron activity (on-transient and off-transient, black arrow heads in the figure). When the pathological gene is expressed, the ERG trace can be used to estimate what part of the visual system is impaired.

**Figure 4.**
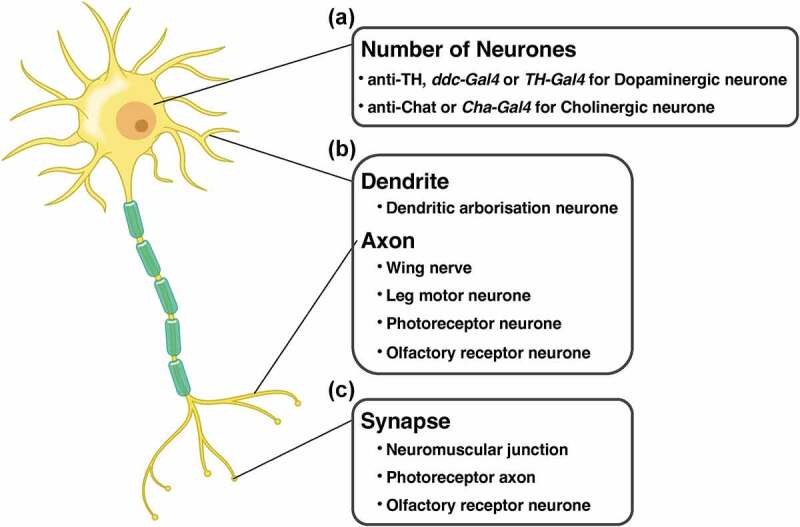
Cell types and experimental systems of *Drosophila* for evaluating the effects of disease protein toxicity on each region in neurone. (A) the number of cell bodies is quantified by visualisation of the dopaminergic or the cholinergic neurones. (B) The degeneration of neurites is evaluated in dendrite (dendritic arborisation neurone) and axons (wing nerve, leg motor neurone, photoreceptor neurone, and olfactory receptor neurone). (C) The number and the structure of synapse is quantified in neuromuscular junction, photoreceptor axon, and olfactory receptor neurone.

‘Humanised’ flies, using the Gal4/UAS system or CRISPR, are often utilised in the study of neurodegenerative diseases in *Drosophila*. However, it is impossible to completely mimic the pathology because the organs of humans and flies are not identical. Therefore, an appropriate selection of evaluation methods is essential for a precise understanding of the toxicity of pathogenic genes and/or alleles. In this section, we introduce the evaluation method of toxicity (Figure 2) and neural function (Figure 3) using fly and the quantification method of each neural area (cell bodies, neurites, and synapses; Figure 4 and Table 2) using the *Drosophila* nervous system (CNS) (Supplemental Table 1).

**Table 2.** The effect of pathological genes on the number of the neurones.

Phenotype	Gene	Gal4	Result	Ref. no.
the number of DA neurons	hαsyn	elav	decreased	28
		Ddc	decreased	28, 29, 43, 139
		TH	decreased	48
		TH	not changed	29
	dParkin	LOF	decreased	42
	dPINK1	LOF	decreased	137
	daux	LOF	decreased	139
	dGBA1B	LOF	not changed	132
the number of Cholinergic neurons	hTau	elav	decreased	52

### 4.1. Evaluation of toxicity

#### 4.1.1. Rough eye phenotype

A simple method for evaluating the toxicity of expressed human genes and alleles is REP. The *Drosophila* eye consists of approximately 700–800 basic units called ommatidia, each consisting of 8 photoreceptor neurones (primary sensory neurones) (Figure 2A), 4 cone cells, and 2 primary pigment cells [117]. Although these ommatidia are usually regularly aligned, they may become disordered if ectopic genes induce morphological differentiation, proliferation, or cell death in the cells that make up the ommatidia, thereby causing morphological abnormalities of the compound eye, such as rough eye (Figure 2B). Gene toxicity is evaluated via a qualitative assessment of the structural abnormalities of the eye. If a structural abnormality of the eye is induced by a human gene, modifier screening can be easily performed using the degree of rescue of the structural abnormality as an indicator. This method does not require any special equipment other than epi-microscopy and it is possible to efficiently evaluate toxicity. However, there are a few disadvantages to the assay. First, since REP reflects developmental disorders in the compound eye, it is not the most suitable model for evaluating the toxicity in age-dependent neurodegeneration. Second, the quantitative evaluation of REP is difficult and has been limited to qualitative evaluation. Recent studies have established methods for quantifying the REP [118,119]. These methods extract the position of each ommatidium from images of compound eyes taken by light microscopy and scanning electron microscopy. By taking advantage of the hexagonal arrangement of normal ommatidia, the disorderliness of each sample is calculated by measuring the distance and angle between each ommatidium. It was difficult to perform large-scale experiments such as screening and detecting slight effects on REP from gene interactions or drugs since the phenotype was judged subjectively by a well-trained observer in the past. However, quantitative methods can perform such analyses automatically and non-biasedly [120,121].

#### 4.1.2. Counting the number of rhabdomeres per ommatidium through pseudopupil assay

Another simple evaluation system for neurotoxicity is the pseudopupil assay [122]. In insects and other organisms with compound eyes, pseudopupil appear as black spots on the surface of the compound eyes. The ommatidium has a straw-shaped structure, and hence its depth can be observed (as a black spot) only when the observer’s eye line coincides with the long axis of the ommatidium (Figure 2C). At this time, the rhabdomere, a signalling compartment located on the apical side of the photoreceptor neurone, of the ommatidium can be observed using a microscope. Usually, there are eight rhabdomeres per ommatidium; however, the rhabdomere of the R8 neurone is located under that of the R7 neurone, and thus seven rhabdomeres can be observed when viewed from outside [123]. Pseudopupil assay can be used to quantitatively evaluate the toxicity of ectopically expressed genes by counting the number of rhabdomeres reduced by the genes. When the number of rhabdomeres is reduced, the expressed human genes may affect photoreceptor survival, differentiation, morphology, and planar polarity [124]. As with REP, it is possible to perform an assay using only a microscope without the need for special equipment. Alternatively, to analyse the structure in more detail, it is possible to visualize the rhabdomere with phalloidin [125], which highlights the F-actin bundles, and observe it with a confocal microscope. Additionally, it is possible to observe the ultrastructure with transmission electron microscopy (TEM). Nevertheless, this method has the disadvantage that only the effects of ectopically expressed gene on the cell body are observed; the effects on neurite components, such as axons and dendrites, cannot be observed.

#### 4.1.3. Measuring the thickness and counting the vacuoles

In the visual system, the analysis of the retina using paraffin sections is frequently used to assess the toxicity of human genes. In *Drosophila*, the retina is a complex of ommatidia, and the length of the long axis of the ommatidial cell body corresponds to the thickness of the retina (Figure 2D). The retinal thickness has been widely used as a quantitative index of neurodegeneration, as the expressions of SCA1 and SCA3/MJD genes, which are causative genes of polyglutamine disease, in the visual system using the *GMR-Gal4* driver reportedly reduced retinal thickness [24,126–128]. The presence of vacuoles in the retina has also long been recognised as a hallmark of degeneration (Figure 2D) [129]. The number and area of vacuoles are known to increase in old flies, flies expressing genes related to neurodegenerative diseases, and flies that sustain head trauma [52,130,131]. However, the retinal is inadequate to perform a more comparable analysis in the human brain. Recent studies have analysed vacuoles in the central brain, a functional analogue in flies of the hippocampus and cerebral cortex, which are vulnerable in human disease [132,133]. Expressing the respective disease genes in disease-specific neuronal species (e.g. dopaminergic neurones in PD and cholinergic neurones in AD) and evaluating the vacuoles in the central brain may reveal the mechanism by which the genes cause neurodegeneration in models more closely mimicking human disease.

In these assays, fly heads embedded in paraffin are cut to a thickness of 4–7 μm and stained with haematoxylin and eosin. Using the prepared specimens, the thickness of the retina and the number and area of the vacuoles are quantified. Therefore, this procedure requires a microtome in addition to a microscope. This technique enables a quantitative evaluation of the degree of degeneration caused by disease-causing proteins and the effects of modifiers on degeneration; nonetheless, it is difficult to obtain reproducible results because the values of the abovementioned parameters vary greatly depending on the angle of sectioning and the location of the retina.

A recent attempt to quantify vacuoles by whole-brain imaging without using paraffin sections has enabled the performance of a precise quantitative analysis of vacuole-like degeneration in each brain region [134]. In the procedure, the whole brain is stained with phalloidin and DAPI is scanned using a confocal microscope or a two-photon laser microscope. By manually measuring the area and number of vacuoles per slice using ImageJ, the authors revealed that repetitive brain trauma increases the number and area of vacuoles in the brain in the long term [131]. Using 3D image analysis software like IMARIS (Bitplane), the method will be able to measure parameters like the number, volume, and shape of each vacuole semi-automatically. In addition, by combining this approach with a variety of cellular dysfunction markers, such as cell death, autophagy, and mitochondrial dysfunction, it will be possible to quantify the location of degenerating cells and what is happening inside them at the whole-brain level.

#### 4.1.4. Life span assay

The Kaplan-Meier survival curve is also commonly used to assess the toxicity of disease genes. To draw the survival curve, the flies that emerged on the same day are collected. Every two or three days, the flies are transferred to a new vial and the number of dead flies is recorded. This is repeated until all flies are dead (Figure 2E). The Gal4/UAS system can be used to express these genes for each neuronal subtypes and measure the survival curve to identify the neurone types that have a critical role in life span. For example, a previous study reported that whole-nerve expression of a pathogenic form of ATXN1, a causative gene of SCA1, reduced life span, but the cholinergic neuron-specific expression did not [135]. These results suggest that cholinergic neurones are resistant to pathogenic alleles of ATXN1. Note that survival curves can only be measured in adults and thus cannot be analysed for flies that die before eclosion.

### 4.2. Methods for evaluating effects of genes on each region of the cell

#### 4.2.1. Quantifying cell bodies

Although the pseudopupil method described above can be used to indirectly quantify the number of cell bodies by counting the photoreceptor rhabdomeres, it is possible to directly quantify the number of cell bodies in the central brain (Figure 4A). Dopaminergic neurons in the substantia nigra are damaged in PD, and thus the number of cell bodies in dopaminergic neurones are usually counted in fly models of PD. There are eight dopaminergic neurone clusters in the central brain of *Drosophila* [136], and the number of cell bodies of five species (PAL, PPL1, PPL2, PPM3, and PPM1/2) are mainly used for quantification [137–139]. The cell body can be visualised using tyrosine hydroxylase (TH) antibody – an enzyme involved in the synthesis of dopamine – and *ddc-gal4* and *TH-Gal4*, which are dopaminergic neurone-specific Gal4 drivers. The cell bodies visualised using these methods are obtained by confocal microscopy or paraffin sectioning, and the number of cell bodies is quantified. Similarly, a study using a fly model of AD have applied cholinergic neurone-specific Gal4 (*Cha-Gal4*) to quantify the number of cholinergic neurone cell bodies [52]. In contrast to dopaminergic neurones, cholinergic neurones do not form distinct clusters. Therefore, the study ectopically expressed *Tau* in cholinergic neurones by *Cha-Gal4* and visualised the neurones by using AT8, a phosphorylated Tau-specific antibody. Finally, they counted the number of cell bodies of cholinergic neurones in a compartment of the optic lamina. In addition to these neurotransmitter-specific labelling methods, Gal4 and antibodies that satisfy the following conditions can be used to quantify the effects of human genes on cell bodies: (1) the number of cell bodies among individuals is relatively stable, and (2) the cell bodies are sparse enough to allow manual counting.

#### 4.2.2. Quantifying neurites

The degeneration of neurites, such as axons and dendrites, is observed in almost all patients with neurodegenerative diseases. The accumulation of degeneration is considered to cause neural function loss in these patients. Therefore, elucidating the molecular basis of neurite degeneration is a major objective of research on neurodegenerative diseases, and is expected to help develop therapies to slow or stop the progression of neurodegenerative diseases. Therefore, an experimental system that can be used to easily evaluate neurite degeneration is required (Figure 4B).

Many systems can be used to observe neurite degeneration in the fly peripheral nervous system (PNS). One such experimental system involves the wing nerve. Taking advantage of the transparency of *Drosophila* wings, axon morphology can be observed without dissection, making it suitable for large-scale experiments such as screening and live imaging [140]. Leg motor neurones constitute an excellent system for examining the effects of human genes on the axons of motor neurones [141]. In practice, a forced expression of *TDP-43* Q331K, a pathogenic allele of TDP-43, induced age-dependent dying-back axonal degeneration in the leg motor neurone; more so, EMS-based forward genetic screening identified several factors associated with the axonal toxicity of TDP-43 [141]. The dendritic arborisation (da) neurone of the fly larva is also suitable for use in observing neurites. The da neurone is classified into four classes (class I to IV) based on the gene expression and dendritic arbitration pattern; moreover, the shape of the da neurone is highly conserved among individuals and is useful for intact neurite observation [142]. Using this experimental system, an ectopic expression of pathogenic SCA3 and SCA1 inhibited the formation of F-actin in the class III and IV da neurones through the Rac-PAK signalling pathway, and affected dendrite maintenance [143].

A quantification method has also been established in these PNS models. In both wing nerve and leg motor neurone models, by combining Mosaic analysis with a repressible cell marker (MARCM) with genetically encoded flippase, it is possible to visualise a few cells, thereby enabling a direct evaluation of the number of axons [141,144]. In the da neurone model, the number of branches and the total length of the dendrites are adequately evaluated. Recently, several software programs have been developed for the automated or semi-automated quantification of these parameters [145–147].

However, these quantitative methods often have to be applied manually or subjectively to determine the degree of degeneration; therefore, it is difficult to conduct large-scale experiments such as screening. Furthermore, no system can hitherto be used to quantitatively evaluate neurite degeneration in the CNS. Recently, we developed a novel method to quantify the number of axons in R7 photoreceptor neurones [148,149]. In this method, samples stained with anti-Chaoptin, which selectively visualises R7 and R8 neurones, were scanned using a confocal microscope; the R7 axon terminals were extracted from the image and counted using IMARIS software [148]. In addition, by combining machine learning image processing with a Python-based counting system, we automated this process, allowing for the quantification of the number of axons at 60 seconds per sample using a typical workstation [149]. Using our system, we found that the number of R7 axons decreased when the representative disease proteins and pathogenic alleles were expressed in a photoreceptor-specific manner. Therefore, by using this system, it is expected that the mechanism of axonal degeneration caused by disease proteins can be elucidated, and the genes and drugs that suppress degeneration can be identified rapidly.

Alternatively, olfactory receptor neurones (ORNs), used as a model for axotomy, can be used to quantify axonal degeneration in the CNS [150]. The ORN is the primary sensory nerve of the olfactory system, which projects axons from the third antenna segment or maxillary palps to the antennal lobe – the primary olfactory centre. The surgical removal of the third antenna segment or maxillary palps causes Wallerian degeneration. Although a direct quantification of the axonal number of ORN is difficult, because of its bundled structure, it is possible to measure the degree of degeneration by evaluating the presence of ORNs in antennal lobes via the visualisation of a limited number of ORNs using the specific Gal4/UAS system.

Compared to axons, there is still no quantitative method for dendritic degeneration in the CNS. If a dendritic quantification method is established, the differences between the degeneration mechanisms in the axons and dendrites will be revealed.

#### 4.2.3 Quantifying synapses

Synapses are minimal units of communication between neurones. Neural information is transmitted through neurotransmitters released from synaptic vesicles at presynaptic terminals located on axons; these neurotransmitters are received by postsynaptic terminals located on the dendrites of specific neighbouring neurones. The region of the presynaptic terminal that releases neurotransmitters is called the active zone (AZ). It is thought that proteins such as voltage-gated Ca^2+^ channels and soluble N-ethylmaleimide-sensitive-factor attachment protein receptors (SNAREs) accumulate in the AZ to actively control neurotransmitter release [151].

Structural or functional abnormalities of presynaptic terminals have been reported in many neurodegenerative diseases and are thought to be early symptoms of these diseases [152,153]. In AD, amyloid precursor protein and its fragment Aβ, which have been suggested to be involved in the pathology of AD, are involved in the formation of synapses and the expression of presynaptic proteins [154,155]. The pathogenic allele of Tau protein, which builds up abnormal forms in the brains of patients with AD, reportedly causes age-dependent synaptic loss [156]. In PD, α-synuclein, a major component of Lewy bodies, is localised at presynapses in healthy individuals. Furthermore, recent studies have shown that it is involved in synaptic vesicle recycling and SNARE protein assembly [157,158]. An overexpression of α-synuclein reduces the recycling pool of synaptic vesicles [159].

Thus, neurodegenerative diseases and synaptic abnormalities are closely linked; consequently, it is essential to visualise and quantify synapses to elucidate their molecular pathomechanisms using a fly model (Figure 4C). In the PNS, the larval NMJ is frequently used as a model for studying synapses. To observe the synapse structure, confocal microscopy or TEM has been employed. On confocal microscopy, neurone membrane is often visualised by staining with Horse Radish Peroxidase (HRP) antibody and the active zone with Bruchpilot (Brp) antibody. In addition to counting the number of boutons, observation by confocal microscope can also count ‘ghost boutons’, immature boutons that do not contain an active zone, and ‘satellite boutons’, boutons that germinate excessively from primary boutons. Therefore, it is possible to evaluate the effects of pathological genes/alleles expressed in NMJ on the synaptogenesis process. In addition, it has recently become possible to semi-automatically quantify the size of the NMJ and the number of AZs using macros available in an open-source image analysis software, Fiji [160]. On TEM, it is possible to observe the ultrastructure of presynaptic cells such as synaptic vesicles and T-bar. The larval NMJ is only a model of the presynaptic cell and, due to its nature, cannot provide post-synaptic biological information. In the CNS, the quantification of AZs has been reported in ORNs and R8 photoreceptor neurones [161,162]. In both models, the AZ was visualised via an ectopic expression of Brp (one of the major components of the AZ) with a fluorescent protein tag; quantification was performed using IMARIS (Bitplane), after the images were scanned with a confocal microscope. Recently, focused ion-beam scanning electron microscopy and expansion lattice light-sheet microscopy have been used to scan the whole brain to reveal connectomes [163]. Using machine learning, pipelines have been developed to extract and quantify synapses from data obtained using the abovementioned microscopy techniques, thereby enabling the quantification of synapses at whole-brain and single-cell resolutions [164]. However, these microscopy techniques require a long time to capture samples; hence, a combination of tagged Brp and confocal microscopy would be appropriate for large-scale experiments such as screening.

### 4.3. Methods for evaluating effects of genes on neural functions

Higher brain functions such as memory, circadian rhythms, and movement are exacerbated in human patients with neurodegenerative diseases. In *Drosophila*, these higher functions are also impaired as well as the degeneration of neural structures. This section presents experimental methods for monitoring neural function in *Drosophila*.

The climbing assay is the easiest and most frequently used method for measuring the motor function of flies (Figure 3A). In this assay, flies are placed in an empty vial, and the vial is tapped to drop the flies to the bottom of the vial. At this point, you can record a vial and calculate the percentage of flies that climbed to a certain height after a certain time. The assay is based on the negative geotaxis of flies. While healthy, young flies immediately start climbing the vial wall when they fall to the bottom, the rate and speed of climbing is reduced in older flies and flies that express a disease gene, such as TDP-43 in their nervous system [69]. A fully automated method for this assay has been reported [165,166], but it can be carried out with a minimum of a vial and a camera, and computational methods for analysing captured video have been developed [167].

Olfactory memory is widely used to measure the memory and learning ability of flies (Figure 3B). It has been reported that the ability is impaired in aged flies and flies that express disease genes in the mushroom body, the olfactory memory center [12,49,168,169]. In flies, memory assays have been well established, using a T-maze device [170]. In this assay, an unconditioned stimulus (US), such as an electric shock (for aversive memory) or sucrose (for appetitive memory), is applied to a constant flow of air with a specific odour (3-octanol and 4-methylcyclohexanol are mostly used) for conditioning. Subsequently, another odour is continuously applied without the US. The two odours used for conditioning are applied to each end of the T-maze for testing. The performance index is calculated based on the number of flies on the two arms of the T-maze at certain times. When many flies are concentrated in the conditioned odour, the PI is positive; when the odour is avoided, it is negative; and when no flies are recalled, the PI is zero. Furthermore, live imaging using the calcium indicator GCaMP has also been performed well, allowing direct monitoring of neuronal activity.

For measuring circadian rhythms and sleep, the *Drosophila* Activity Monitor (DAM) system is useful. In the assay, flies are placed in a thin glass tube filled with food at one end and set in the device. The device emits infrared light perpendicularly to the tube, and whenever a fly in the tube crosses the infrared light, it is counted as a ‘move’. A graph of activity by time is called an actogram (Figure 3C). In normal flies, activity peaks are observed in the morning and evening during the standard light-dark cycle (light: 12 hours; dark: 12 hours) [171]. Sleep is often defined as the absence of movement for more than 5 minutes; hence it is possible to measure sleep length using this system [172]. Circadian rhythm disorder and decreased sleep time have been reported in many disease model flies [37,48,135,173–175].

In addition, there are some experimental systems which mimic pathology. Seizure is more likely to occur in patients with many neurodegenerative diseases, and this is possible to mimic in flies. A mechanically induced seizure method called bang-sensitivity assay is frequently used due to its simplicity [176]. In this assay, flies in a vial are subjected to a vortex (the stimulus is called ‘Bang!’) for 10 seconds, and their behaviour is observed. Young and healthy flies show no changes in behaviour or position when subjected to bang-stimulation. In contrast, the fruit fly with higher seizure susceptibility turns over and recovers through initial seizure-like behaviour, paralysis, and recovery seizure stages. Seizure susceptibility is evaluated by quantifying the proportion of flies that recover over time and that of flies with seizures. Some fly mutants showing higher seizure susceptibility have mutations in the genes involved in mitochondrial function, which is consistent with the high frequency of epilepsy in human mitochondrial disorders [177]. Increased seizure susceptibility has also been reported in many disease model flies [132,133,178].

For direct observation of neural activity, EJPs and electroretinogram (ERG) are frequently used as fast and easy methods. Typically, EJPs recordings are performed in larval NMJs, in which the suction electrode stimulates the nerve and the EJPs are recorded in the muscle side microelectrode. EJPs reflect overall NMJ activity, and their amplitude and decay can estimate the amount of neurotransmitter release and receptor activity in the muscle. In ERG recording, a reference electrode is inserted into a thorax, and a recording electrode is placed on the surface of the compound eye to record the compound field potentials from photoreceptors and downstream neurones in the visual system during light flashes [179,180]. An ERG trace consists of four phases: transient spikes at the onset and offset of light flash (called on-transient and off-transient, respectively), depolarisation during light stimulation, and repolarisation after light stimulation ends (Figure 3D). The on-transient and off-transient correspond to the potential of the postsynaptic neurone receiving the signal from the photoreceptor neurone. Depolarisation and repolarisation reflect the activation and inactivation of the photoreceptor neurone, respectively [181]. Thus, abnormal on-transient and off-transient spikes indicate malformation or dysfunction of synapses, and defects in depolarisation and repolarisation indicate structural abnormalities of photoreceptors and defects in phototransduction. In compound eyes, mosaic analysis can be easily performed using the FLP/FRT system. Therefore, it is possible to evaluate the effects on the neural function of disease genes that are lethal when expressed in the whole body or the whole nervous system. Several disease models have been reported to exhibit the alterations of ERG, and some of these studies have validated the structural defects of synapse using TEM [182,183].

### 4.4. Future direction in selecting appropriate evaluation methods

The classical quantification methods that are still widely used today are simple, although they have quantification- and reproducibility-related limitations. In recent years, the development of algorithms and machine learning have made it possible to perform more quantitative evaluations [149,184]. It is also critical to determine the tissues that are used to evaluate the effects of human genes. In fact, we found that the REP and axon phenotypes did not coincide when the human disease gene was overexpressed, despite using the same Gal4 driver [149]. These results imply that observing REP alone would cause a misinterpretation of the axonal toxicity of the disease-causing gene. By combining appropriate experimental systems with appropriate quantification methods, studies on neurodegenerative diseases in flies will provide more precise insights into the pathogenesis of neurodegeneration.

## 5. Conclusions

*Drosophila* has been used to study various neurodegenerative diseases by searching for modifier genes for disease pathology. Furthermore, various methods have been developed to more accurately assess neurodegeneration. From large-scale gene exploration, new pathological mechanisms can be identified by rapid genetic and molecular pathway analyses. This approach is unique to simple models, such as *D. melanogaster, C. elegans*, and *S. cerevisiae*, and can be a different angle of breakthrough from studies using human and vertebrate models. Based on the knowledge obtained from simple model organisms, it is possible to efficiently verify disease mechanisms in vertebrate models and humans.

Simple model organisms have fewer conserved genes and molecular pathways than vertebrate models such as zebrafish, mice, and marmosets. It is also difficult to develop models that mimic diseases at the organ or tissue level. These are the limitations of using simple model organisms. The benefits and limitations of simple model organisms will be better understood and the researches on neurodegenerative diseases using a combination of flies, vertebrate models, and humans will increase in the future.

## Supplementary Material

Supplemental Material
